# Oligohydramnios: a prospective study of fetal, neonatal and maternal outcomes in low-middle income countries

**DOI:** 10.1186/s12978-020-0854-y

**Published:** 2020-01-30

**Authors:** Lester Figueroa, Elizabeth M. McClure, Jonathan Swanson, Robert Nathan, Ana L. Garces, Janet L. Moore, Nancy F. Krebs, K. Michael Hambidge, Melissa Bauserman, Adrien Lokangaka, Antoinette Tshefu, Waseem Mirza, Sarah Saleem, Farnaz Naqvi, Waldemar A. Carlo, Elwyn Chomba, Edward A. Liechty, Fabian Esamai, David Swanson, Carl L. Bose, Robert L. Goldenberg

**Affiliations:** 1 Instituto de Nutrición de Centro América y Panamá (INCAP), Guatemala City, Guatemala; 2 Social Statistical and Environmental Health Sciences, RTI International, Durham, NC USA; 3 Department of Radiology, University of Washington Medical Center, Seattle, WA USA; 4 Department of Pediatrics, University of Colorado, Denver, CO USA; 5 Department of Pediatrics, University of North Carolina School of Medicine, Chapel Hill, NC USA; 6 Kinshasa School of Public Health, Kinshasa, Democratic Republic of the Congo; 7 Department of Radiology, Aga Khan University, Karachi, Pakistan; 8 Department of Community Health Sciences, Aga Khan University, Karachi, Pakistan; 9 Department of Pediatrics, University of Alabama at Birmingham, Birmingham, AL USA; 10 Department of Pediatrics, University of Zambia, Lusaka, Zambia; 11 Department of Pediatrics, Indiana University, Indianapolis, IN USA; 12 School of Medicine, Moi University, Eldoret, Kenya; 13 Department of Radiology, Harborview Medical Center, University of Washington Medical Center, Seattle, WA USA; 14 Department of Obstetrics/Gynecology, Columbia University, New York, NY USA

**Keywords:** Oligohydramnios, Low and middle-income countries, Ultrasound, Pregnancy outcomes

## Abstract

**Background:**

Oligohydramnios is a condition of abnormally low amniotic fluid volume that has been associated with poor pregnancy outcomes. To date, the prevalence of this condition and its outcomes has not been well described in low and low-middle income countries (LMIC) where ultrasound use to diagnose this condition in pregnancy is limited. As part of a prospective trial of ultrasound at antenatal care in LMICs, we sought to evaluate the incidence of and the adverse maternal, fetal and neonatal outcomes associated with oligohydramnios.

**Methods:**

We included data in this report from all pregnant women in community settings in Guatemala, Pakistan, Zambia and the Democratic Republic of Congo (DRC) who received a third trimester ultrasound as part of the First Look Study, a randomized trial to assess the value of ultrasound at antenatal care. Using these data, we conducted a planned secondary analysis to compare pregnancy outcomes of women with to those without oligohydramnios. Oligohydramnios was defined as measurement of an Amniotic Fluid Index less than 5 cm in at least one ultrasound in the third trimester. The outcomes assessed included maternal morbidity and fetal and neonatal mortality, preterm birth and low-birthweight. We used pairwise site comparisons with Tukey-Kramer adjustment and multivariable logistic models using general estimating equations to account for the correlation of outcomes within cluster.

**Results:**

Of 12,940 women enrolled in the clusters in Guatemala, Pakistan, Zambia and the DRC in the First Look Study who had a third trimester ultrasound examination, 87 women were diagnosed with oligohydramnios, equivalent to 0.7% of those studied. Prevalence of detected oligohydramnios varied among study sites; from the lowest of 0.2% in Zambia and the DRC to the highest of 1.5% in Pakistan. Women diagnosed with oligohydramnios had higher rates of hemorrhage, fetal malposition, and cesarean delivery than women without oligohydramnios. We also found unfavorable fetal and neonatal outcomes associated with oligohydramnios including stillbirths (OR 5.16, 95%CI 2.07, 12.85), neonatal deaths < 28 days (OR 3.18, 95% CI 1.18, 8.57), low birth weight (OR 2.10, 95% CI 1.44, 3.07) and preterm births (OR 2.73, 95%CI 1.76, 4.23). The mean birth weight was 162 g less (95% CI -288.6, − 35.9) with oligohydramnios.

**Conclusions:**

Oligohydramnos was associated with worse neonatal, fetal and maternal outcomes in LMIC. Further research is needed to assess effective interventions to diagnose and ultimately to reduce poor outcomes in these settings.

**Trial registration:**

NCT01990625.

## Plain English summary

Low levels of amniotic fluid (also known as oligohydramnios) have been associated with a number of adverse pregnancy outcomes in high-income countries. In this analysis of data from pregnancies in the First Look Trial from Guatemala, Pakistan, Zambia and the Democratic Republic of Congo involving nearly 13,000 women with a third trimester ultrasound examination, oligohydramnios was found in about 1 in 150 pregnancies. Oligohydramnios was associated with higher rates of maternal hemorrhage, fetal malposition and cesarean delivery than in pregnancies without oligohydramnios. Higher rates of poor fetal/neonatal outcomes were also associated with oligohydramnios, including a 5-fold increase in stillbirths and a 3-fold increase in deaths among babies less than 28 days of age. The babies were also twice as likely to be born prematurely or to be low birth weight (weigh less than 2500 g). The babies from pregnancies complicated by oligohydramnios weighed on average 162 g less than those from pregnancies without oligohydramnios. In summary, similar to results from high-income countries, in the low- and middle-income countries studied, oligohydramnios was associated with a number of pregnancy-related complications for the mother and her fetus and newborn.

## Background

An appropriate volume of amniotic fluid is one of the most important components of a healthy pregnancy, as it acts as a protective cushion for the fetus, prevents compression of the umbilical cord, and promotes fetal lung development [1]. While the average volume of amniotic fluid varies with gestational age, abnormally low amniotic fluid volume has been associated with adverse pregnancy outcomes. Oligohydramnios, in which the volume of amniotic fluid is abnormally low (< 500 ml) between the 32nd and 36th weeks of pregnancy, is a serious condition for the fetus and the mother [1, 2]. Oligohydramnios can be diagnosed with ultrasound performed during the late second trimester or the third trimester and is defined by an Amniotic Fluid Index (AFI) below 5 cms or below the 5th percentile to approximate the amniotic fluid volume [3, 4].

In settings where ultrasound use is widespread, rates of oligohydramnios have been reported between 0.5 and 8% among pregnant women [5]. When associated with a fetal anomaly, oligohydramnios is present in as many as 37% of pregnancies and is higher with other pregnancy complications [6]. However, because ultrasound is not commonly used during routine prenatal care in many low and middle-income country (LMIC) settings, the population rates of oligohydramnios and the associated outcomes in LMIC settings are largely unknown.

Maternal conditions such as utero-placental insufficiency, hypertension, preeclampsia, diabetes, chronic hypoxia, rupture of amniotic membranes, dehydration and post-term gestation have been associated with oligohydramnios [1, 2]. Anomalies of the kidneys including congenital absence of renal tissue, obstructive uropathy or decreased renal perfusion also may be contributing factors [7]. Most oligohydramnios cases, however, are idiopathic [1, 2].

Fetal health can be seriously compromised by oligohydramnios, with complications such as pulmonary hypoplasia, meconium aspiration syndrome, fetal compression and, in cases of prolonged rupture of membranes, infections [1, 2, 8, 9]. Women with oligohydramnios are more likely to have an infant with low birth weight [10–13]. In terms of burden of care, higher rates of cesarean delivery for fetal distress and neonatal admission to the intensive care unit have also been associated with oligohydramnios [4, 8]. Timely identification and treatment have been associated with improvement in some maternal and fetal/neonatal outcomes. When detected, clinical management of women with oligohydramnios can include amnioinfusion, early induction of labor and even cesarean delivery [13, 14]. However, gaps in knowledge remain, including the incidence of oligohydramnios in LMIC, the role of the underlying conditions associated with oligohydramnios and their association with oligohydramnios and adverse pregnancy outcomes [15–17].

To address this need, we conducted a secondary analysis of data from the First Look Trial, which aimed to determine if the introduction of ultrasound examinations during antenatal care in low-resource settings improved maternal mortality, maternal near-miss mortality, stillbirth and neonatal mortality. The methods and results of the parent trial have been published [17, 18]. Our objectives in conducting this planned secondary analysis included determining the prevalence of oligohydramnios, risk factors for this condition, and the maternal and fetal outcomes associated with oligohydramnios in LMIC settings.

## Methods

We evaluated oligohydramnios among women enrolled in the First Look Trial, a multi-country cluster randomized study that enrolled pregnant women in rural areas within Guatemala, Pakistan, Kenya, Zambia and the Democratic Republic of Congo (DRC). Briefly, as part of the trial, at each site, medical officers, nurses, midwives and radiographers with no prior ultrasound experience were trained to perform basic obstetric ultrasound examinations to determine gestational age and screen for high-risk conditions. All sonographers received standardized training using the Basic Obstetric Ultrasound Training methodology developed by the University of Washington (UW) team [19–21]. This training consisted of an intensive two-week training led by the UW team with both didactic and hands-on components. In addition, during the next 3 months, a minimum of five examinations were observed directly by an experienced sonographer and all ultrasound examinations, including the images and interpretation, were evaluated by a senior radiologist either at UW or at the site for quality assurance (QA) [20]. Using the web-based application, all ultrasound images were uploaded at the site, then reviewed by the senior QA radiologist with feedback provided to the field sonographers on a regular basis [20, 21]. Throughout the trial, quality control procedures were used to assess and maintain a high rate of accuracy for the ultrasound diagnoses. We emphasize that all sites used the same equipment and that the criteria for diagnosing oligohydramnios were the same for all sites.

For this analysis, we included those participants who had at least one ultrasound examination in the third trimester. We defined oligohydramnios as an amniotic fluid index below 5 cms on one or more ultrasound examinations performed after 28 weeks. All cases of oligohydramnios were confirmed by the central QA team of experienced radiologists at the UW. In addition, approximately 10% of all ultrasound examinations other than those with oligohydramnios were also reviewed for accuracy. Body mass index (BMI) was defined as the mother’s weight in kilograms divided by her height in meters squared. All maternal and infant outcomes up to 6 weeks postpartum were collected by the Global Network’s Maternal Newborn Health Registry [22]. We excluded women from the analysis who were lost to follow-up prior to delivery, maternal deaths that occurred before 20 weeks, and women who had a miscarriage or a medical termination of pregnancy. Because the Kenyan site had no cases of oligohydramnios identified in the third trimester, we present data only from Pakistan, the DRC, Guatemala and Zambia. However, the results were similar with and without the Kenyan data.

Data were keyed and edit checks conducted locally before data were transferred through encrypted transmission to a central data center. We reported pairwise mean differences of oligohydramnios for each site and *p*-values with a Tukey-Kramer adjustment for multiple comparisons from a logistic model adjusting for site using generalized estimating equations (GEE) to account for the correlation of oligohydramnios within cluster. To determine maternal characteristics associated with oligohydramnios, p-values were obtained from logistic models using GEE and adjusting for site and each maternal characteristic. In addition, odds ratios and 95% confidence intervals for delivery complications and fetal/neonatal outcomes were obtained from logistic models, adjusting for oligohydramnios, study site and prior live birth using GEE to account for the correlation of outcomes within cluster.

## Ethics

This study was reviewed and approved by the institutional review boards of participating institutions (Aga Khan University, Pakistan; Moi University, Kenya; University of Zambia; INCAP, Guatemala; and Kinshasa School of Public Health, DRC; University of Washington, Seattle WA; RTI International, Durham NC). All women provided informed consent prior to enrollment in the trial.

## Results

**Fig. 1:**
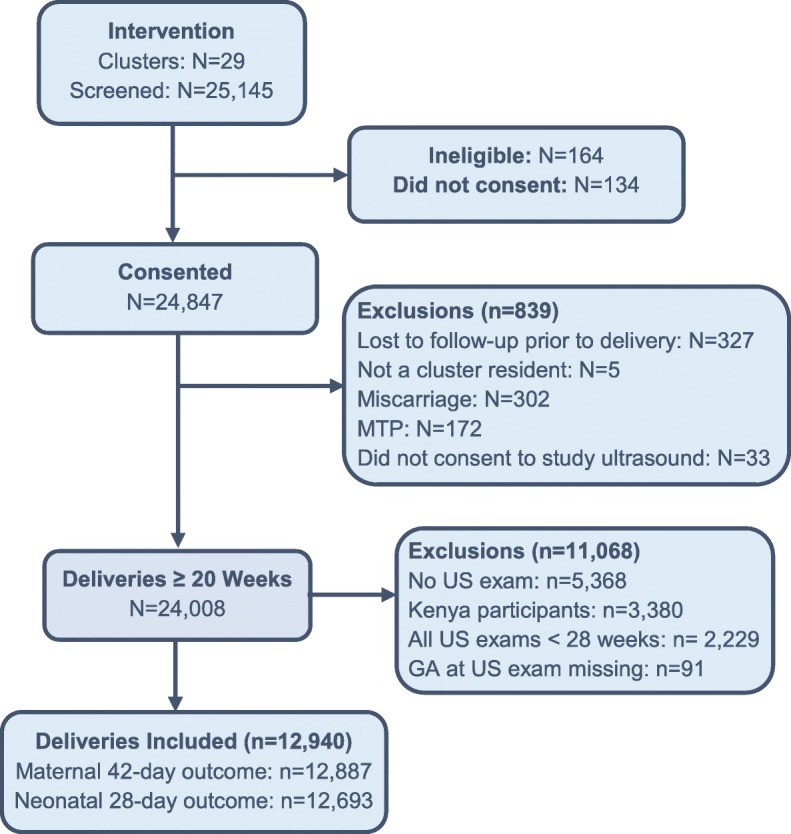
CONSORT diagram

A total of 12,940 participants in Guatemala, Pakistan, Zambia and the DRC received at least one third trimester study ultrasound examination (Fig. 1). Eighty-seven cases of oligohydramnios, equivalent to 0.7% of the subjects in this analysis, were detected on a third trimester ultrasound. Prevalence of oligohydramnios varied among study sites with the lowest rates in Zambia and the DRC (0.2%) and the highest in Pakistan (1.5%) (Table 1). We found differences in prevalence to be statistically significant between the Guatemalan and Pakistan sites that had the highest prevalence, in comparison to the Zambian site that had the lowest prevalence. Mean gestational age at the time of first diagnosis of oligohydramnios was 35.5 ± 4.1 weeks.

**Table 1:** Incidence of oligohydramnios by FIRST LOOK study site

	Overall	DRC	Zambia	Guatemala	Pakistan
At least one US exam ≥28 weeks, n	12,940	1978	3571	5507	1884
Incidence of oligohydramnios, n (%)	87 (0.7)	4 (0.2)	6 (0.2)	49 (0.9)	28 (1.5)
GA at first diagnosis, Mean (sd)	35.5 (4.1)	37.8 (1.4)	35.9 (1.3)	36.6 (3.7)	33.0 (4.3)

The only significant difference in the maternal characteristics between those women with and without oligohydramnios using a logistic regression model with primiparas in the model was found in women with a previous live birth. There were no statistically significant differences among the other maternal characteristics including in the distribution of maternal age, education, parity, maternal height, weight and BMI between participants with or without oligohydramnios (Table 2).

**Table 2:** Maternal characteristics in women with and without oligohydramnios

	With Oligohydramnios	No Oligohydramnios	Overall	*p*-Value^1^
At least one US exam ≥28 weeks, n	87	12,853	12,940	
Maternal age (years), n (%)	87	12,849	12,936	0.2766
< 20	17 (19.5)	2185 (17.0)	2202 (17.0)	
20–35	61 (70.1)	9577 (74.5)	9638 (74.5)	
> 35	9 (10.3)	1087 (8.5)	1096 (8.5)	
Maternal education, n (%)	87	12,851	12,938	0.1698
No formal schooling	30 (34.5)	3209 (25.0)	3239 (25.0)	
Primary	37 (42.5)	4403 (34.3)	4440 (34.3)	
Secondary	19 (21.8)	4873 (37.9)	4892 (37.8)	
University	1 (1.1)	366 (2.8)	367 (2.8)	
Parity, n (%)	82	12,660	12,742	0.1253
0	25 (30.5)	2993 (23.6)	3018 (23.7)	
1	14 (17.1)	2847 (22.5)	2861 (22.5)	
2+	43 (52.4)	6820 (53.9)	6863 (53.9)	
Previous live birth among multipara, n/N (%)	52/57 (91.2)	9092/9667 (94.1)	9144/9724 (94.0)	0.5814
Previous live birth with primipara in denominator, n/N (%)	52/82 (63.4)	9092/12,660 (71.8)	9144/12,742 (71.8)	0.0421
Maternal height, Mean (sd)	151.1 (7.6)	153.5 (8.1)	153.5 (8.1)	0.7097
Maternal weight, Mean (sd)	55.1 (10.0)	55.9 (10.1)	55.9 (10.1)	0.0713
Maternal BMI, Mean (sd)	24.2 (4.2)	23.8 (4.2)	23.8 (4.2)	0.1007

^1^*P*-value from a logistic regression model for at least one oligohydramnios finding adjusting for site and maternal characteristics using general estimating equations to account for the correlation of outcomes within cluster

Women with oligohydramnios had significantly higher incidences of hemorrhage (5.7% vs. 1.7%, OR 2.94, 95% CI 1.31, 6.61) and fetal malposition (5.7% vs. 1.9%, OR 2.44, 95% CI 1.07, 5.59) (Table 3). Cesarean deliveries were more commonly performed in women with oligohydramnios compared to those without oligohydramnios (28.7% vs. 13.5%, OR 2.07, 95% CI 1.41, 3.03). While hypertensive disorders were more common in women with oligohydramnios, 4.6% compared to 2.2%, we were not able to get the model to converge, likely due to the low prevalence of hypertension in the African sites. There were no maternal deaths among the women with oligohydramnios.

**Table 3:** Delivery complications and fetal/neonatal outcomes in women with and without oligohydramnios

	With Oligohydramnios	No Oligohydramnios	Overall	Odds Ratio^b^ or Mean Difference^c^ (95% CI)
Delivery complications				
Obstructed labor, n/N (%)	5/87 (5.7)	531/12,852 (4.1)	536/12,939 (4.1)	0.84 (0.36, 1.93)
Hemorrhage, n/N (%)	5/87 (5.7)	212/12,666 (1.7)	217/12,753 (1.7)	2.94 (1.31, 6.61)
Hypertensive disorder^d^, n/N (%)	4/87 (4.6)	277/12,851 (2.2)	281/12,938 (2.2)	–
Fetal malposition, n/N (%)	5/87 (5.7)	243/12,852 (1.9)	248/12,939 (1.9)	2.44 (1.07, 5.59)
C-section delivery, n/N (%)	25/87 (28.7)	1736/12,851 (13.5)	1761/12,938 (13.6)	2.07 (1.41, 3.03)
Maternal death < 42 days^d^, n/N (rate/100,000 deliveries)	0/87 (0)	12/12,800 (94)	12/12,887 (93)	–
Maternal sepsis, n/N (%)	1/87 (1.1)	134/12,720 (1.1)	135/12,807 (1.1)	0.82 (0.19, 3.57)
Fetal/Neonatal outcomes^a^				
Stillbirth, n/N (rate/1000)	7/87 (80.5)	192/12,852 (14.9)	199/12,939 (15.4)	5.16 (2.07, 12.85)
Male, n/N (%)	43/86 (50.0)	6578/12,849 (51.2)	6621/12,935 (51.2)	0.99 (0.69, 1.41)
Low birth weight, n/N (%)	26/87 (29.9)	1507/12,849 (11.7)	1533/12,936 (11.9)	2.10 (1.44, 3.07)
Multiple gestation, n/N (%)	2/87 (2.3)	76/12,851 (0.6)	78/12,938 (0.6)	1.93 (0.26, 14.37)
Congenital anomaly^d^, n/N (%)	2/77 (2.6)	18/12,513 (0.1)	20/12,590 (0.2)	–
Birth weight, Mean (sd)	2710 (595)	2971 (441)	2969 (443)	−162.3 (−288.6, −35.9)
GA at delivery, Mean (sd)	37.7 (2.9)	38.6 (2.0)	38.6 (2.0)	−0.53 (−1.07, 0.00)
Preterm Birth, n/N (%)	27/85 (31.8)	1438/12,566 (11.4)	1465/12,651 (11.6)	2.73 (1.76, 4.23)
Neonatal death < 28 days, n/N (rate/1000)	6/80 (75.0)	211/12,613 (16.7)	217/12,693 (17.1)	3.18 (1.18, 8.57)

^a^Fetal/Neonatal outcomes are calculated at the maternal level if at least one fetus/neonate has the outcome

^b^Odds ratios from a multivariable logistic regression model adjusting for at least one oligohydramnios finding, previous

live birth and site using general estimating equations to account for the correlation of outcomes within cluster

^c^Mean difference from a multivariable regression model adjusting for at least one oligohydramnios finding and site, previous live birth and site using general estimating equations to account for the correlation of outcomes within cluster

^d^Model did not converge

We also found unfavorable fetal and neonatal outcomes among women with oligohydramnios. Women with oligohydramnios compared to those without had higher risk for stillbirths (80.5 per 1000 births vs. 14.9 per 1000 births, OR 5.16, 95% CI 2.07, 12.85), neonatal deaths within 28 days (75.0 vs 16.7 per 1000 live births, OR 3.18, 95% CI 1.18, 8.57), low birth weight (29.9% vs 11.7%, OR 2.10, 95% CI 1.44, 3.07) and preterm birth (31.8% vs 11.4%, OR 2.73, 95% CI 1.76, 4.23). Congenital anomalies were more common among the offspring of women with oligohydramnios compared to without oligohydramnios (2.6% vs. 0.1%, respectively) but likely due to small numbers, the logistic regression model did not converge. The mean birth weight was significantly lower in the oligohydramnios group based on the model, with a mean difference of − 162.3 g (95% CI − 288.6 g, − 35.9 g).

## Discussion

The overall prevalence of oligohydramnios on a third trimester ultrasound examination performed on average around 35 weeks of pregnancy was 0.7% across sites, with the lowest incidence in Zambia and the DRC (0.2%) and highest in Pakistan (1.5%). These rates are within the ranges found in high-income countries and provide evidence regarding the rate of oligohydramnios in LMIC settings [4, 8–12].

We found no substantial demographic differences among women with or without this condition. However, we did find significant differences in delivery complications; hemorrhage, fetal malposition and cesarean section were significantly more common in women with oligohydramnios. The higher rates of these complications have been noted in studies from high-income countries. Most interesting were the fetal and neonatal outcomes associated with oligohydramnios. The stillbirth rate was five-fold higher and the neonatal death rate three-fold higher in this group. The mean birth weight was lower in women with oligohydramnios by 162 g and the incidences of low birth weight and preterm birth were higher. Similar results have been found in studies from high-income settings [4, 8, 9, 11–13]. During the parent study we emphasized appropriate referral and hospital care for conditions diagnosed by ultrasound including oligohydramnios. However, care at many of the study hospitals was less than optimal and we do not know if better care for women with oligohydramnios and their neonates would have improved the outcomes.

The strengths of the study included the large sample size, more than 12,900 pregnant women had a third trimester ultrasound examination. In addition, we had broad representation with women from 4 countries on 3 continents included in this analysis. The data were all collected prospectively. Every case in which oligohydramnios was diagnosed was also confirmed by a radiologist with extensive expertise in ultrasonography in pregnancy [18–21]. Outcome data were collected independently from the ultrasound study team as part of an ongoing pregnancy outcome registry.

Potential weaknesses included the fact that the sonographers were recently trained and had limited ultrasound experience, although they received excellent training and their examinations were monitored during the study. The timing of the stillbirth was not routinely collected so whether the stillbirth preceded the diagnosis of oligohydramnios or followed it is unclear. There were few congenital anomalies in the oligohydramnios group, so further study of this issue was impractical. While there was little evidence of membrane rupture at the time of the diagnosis of oligohydramnios, routine testing for membrane rupture was not done at that time. The potential reasons for the lower reported rates of oligohydramnios in the African sites compared to the Guatemalan and Pakistan sites are unexplained; however, this discrepancy may suggest that some women with oligohydramnios were missed. We emphasize, however, that every examination diagnosed as having oligohydramnios was confirmed by the QA radiologist. We also emphasize that since data for this analysis came from four countries on three continents, and included 87 cases of third trimester oligohydramnios, we believe the maternal, fetal and neonatal outcomes associated with oligohydramnios are generalizable to many LMIC.

## Conclusions

The incidence of oligohydramnios in our LMIC was not generally associated with the maternal demographic characteristics assessed, but oligohydramnios was associated with a variety of materrnal, fetal and neonatal adverse outcomes. While this study demonstrated that newly trained sonographers were capable of diagnosing oligohydramnios [19–22] and that women with oligohydramnios often had worse outcomes than women without oligohydramnios, our data do not prove that diagnosing oligohydramnios during pregnancy with ultrasound improves outcomes.

Some studies from high income countries suggest that treating some cases of oligohydramnios may improve certain outcomes [13, 14, 23], but whether interventions such as amnioinfusion or early delivery or delivery by cesarean section would achieve similar results in LMICs is unknown [24, 25]. The overall trial showed no benefit of ultrasound for any important outcome including maternal death or near-miss maternal mortality, stillbirth and neonatal mortality [19]. However, since the main trial was not specifically aimed at improving outcomes associated with oligohydramnios, the benefit of these interventions in LMIC, if any, remains unknown.

## Data Availability

The dataset analysed during the current study are available at NICHD Data and Specimen Hub (NDASH) (https://dash.nichd.nih.gov/).

## Abbreviations

LMIC: Low-middle income countries
US: Ultrasound
